# Impact of proper surgical treatment on the survival of patients with epithelial ovary cancer

**DOI:** 10.1590/0100-6991e-20223231-en

**Published:** 2022-11-10

**Authors:** FLAVIO HENRIQUE FARIA, ANA PAULA DRUMMOND LAGE, ANGÉLICA NOGUEIRA RODRIGUES, ALBERTO JULIUS ALVES WAINSTEIN

**Affiliations:** 1 - Faculdade Ciências Médicas de Minas Gerais, Pós-Graduação - Belo Horizonte - MG - Brasil

**Keywords:** Ovarian Neoplasms, Carcinoma, Ovarian Epithelial, Cytoreduction Surgical Procedures, Neoplasm Staging, Neoplasias Ováricas, Carcinoma Epitelial Ovárico, Procedimentos Cirúrgicos de Citorredução, Estadiamento de Neoplasias

## Abstract

**Objective:**

to evaluate the quality of surgical treatment of ovarian cancer patients and assess the impact of adequate surgical oncological treatment on disease-free survival and overall survival of patients with advanced epithelial ovarian cancer.

**Methods:**

this is an observational, retrospective study with quantitative analysis, with the collection of data in medical records of a temporal convenience sample of patients diagnosed with ovarian cancer admitted to a High Complexity Oncology Unit, in Belo Horizonte, from the period of 2014 to 2020.

**Results:**

a total of 91 patients diagnosed with ovarian cancer were evaluated, with the epithelial histopathological type being the most frequent (85%). Of this total, 68 patients (74.7%) had advanced-stage ovarian cancer. Appropriate surgical treatment was performed in 30.9% of patients with advanced epithelial ovarian cancer and the type of performed surgery was statistically significant for overall survival. This low proportion of appropriate surgical oncological treatment was not related to surgical specially or surgeon competence, but mainly to advanced disease related to patient flow at UNACON. It was not possible to confirm if the advanced-stage disease was related to tumor biology or losing time from diagnosis to oncological surgery.

**Conclusion:**

overall survival of advanced-stage epithelial ovarian cancer patients is directly influenced by appropriate surgical treatment, however, in this study, the percentage of advanced ovarian cancer receiving adequate surgical treatment was much lower than the rates reported in the literature. To improve these outcomes, we believe that surgeons should keep following patients during neoadjuvant chemotherapy to point to a better time for surgery, and clinical oncologists should better consider adequate oncological surgery as one of the pillars of ovarian cancer treatment and get more involved in facilitating surgeries.

## RESUMO

**Objetivo:**

avaliar a qualidade do tratamento cirúrgico de pacientes com câncer de ovário e o impacto do tratamento oncológico cirúrgico adequado na sobrevida livre de doença e sobrevida global de pacientes com câncer de ovário epitelial avançado.

**Métodos:**

Trata-se de um estudo observacional, retrospectivo, de análise quantitativo, com coleta de dados em prontuários de uma amostra de conveniência temporal de pacientes com diagnóstico de câncer de ovário internadas em uma Unidade de Oncologia de Alta Complexidade (UNACON), em Belo Horizonte, no período de 2014 a 2020.

**Resultados:**

foram avaliadas 91 pacientes diagnosticadas com câncer de ovário, sendo o tipo histopatológico epitelial o mais frequente (85%). Desse total, 68 pacientes (74,7%) apresentavam câncer de ovário em estágio avançado. O tratamento cirúrgico adequado foi realizado em 30,9% das pacientes com câncer de ovário epitelial avançado e o tipo de cirurgia realizada foi estatisticamente significativo para a sobrevida global. Essa baixa proporção de tratamento cirúrgico oncológico adequado não esteve relacionada à especialidade cirúrgica ou competência do cirurgião, mas principalmente à doença avançada relacionada ao fluxo de pacientes na UNACON. Não foi possível confirmar se a doença em estágio avançado estava relacionada à biologia tumoral ou à perda de tempo do diagnóstico para a cirurgia oncológica.

**Conclusão:**

A sobrevida global de pacientes com câncer de ovário epitelial em estágio avançado é diretamente influenciada pelo tratamento cirúrgico adequado. Porém, o percentual de câncer de ovário avançado recebendo tratamento cirúrgico adequado foi muito inferior aos índices relatados na literatura. Para melhorar esses resultados, acreditamos que os cirurgiões devem continuar acompanhando as pacientes durante a quimioterapia neoadjuvante para apontar um melhor momento para a cirurgia, e os oncologistas clínicos devem considerar melhor a cirurgia oncológica adequada como um dos pilares do tratamento do câncer de ovário e se envolver mais na facilitação das cirurgias.

## INTRODUCTION

Epithelial ovarian cancer is the most prevalent histopathological type (85 to 90%) among malignant ovarian tumors^1^ ^,^ ^2^. It is considered the leading cause of death among gynecological cancers and the fifth leading cause of cancer death in women. It is more prevalent in the sixth and seventh decade of life, with more than 70% of patients being diagnosed in advanced stages of the disease, leading to an average survival in 5 years, which ranges from 30.3% to 44.1% and less than a 40% chance of cure. In 2018, in the United States, it was estimated that 22,240 new diagnostic cases and 14,070 deaths were due to this pathology^2^ ^-^ ^5^.

In clinical practice, patients with epithelial ovarian cancer are divided into two large groups according to the staging system of the International Federation of Gynecology and Obstetrics (FIGO): patients with early-stage disease (FIGO stage I-IIA) and patients with advanced-stage disease (FIGO stage IIB-IV)^6^ ^-^ ^8^. The five-year overall survival is inversely related to disease staging, falling from almost 90% in FIGO stage I to approximately 20% in FIGO stage IV^8^.

As treatment options for these patients, a combination of surgery and chemotherapy should be always available, and the sequence of using these therapies depends on the extent, volume, and location of the disease; associated with the patient’s clinical conditions (performance status, comorbidities and/or medical contraindications)^7^ ^-^ ^10^.

The most important prognostic factors in epithelial ovarian cancer are staging, the degree of histopathological differentiation, and the volume of disease remaining after surgical treatment. This last factor is the only one that can be controlled by the medical team^2^ ^,^ ^7^ ^-^ ^10^.

The primary cancer treatment for ovarian cancer consists of staging surgery and/or cytoreductive surgery, followed, in most patients, by systemic chemotherapy^7^ ^-^ ^10^.

Cytoreductive surgery is indicated, as initial treatment, in patients with advanced-stage ovarian cancer. The main objective is total resection (complete debulking) or almost total resection when macroscopic residual lesions smaller than 1cm in their largest diameters persist intra-abdominally (optimal debulking). Complete and/or optimal debulking rates of up to 70% to 80% have been reported in several reference centers in the treatment of ovarian cancer, however, over 50% rates are considered acceptable in the literature^7^ ^-^ ^11^.

In cases of patients with high-risk complications for a major surgical procedure or whose disease extension converts it impossible to perform complete or optimal cytoreductive surgery, neoadjuvant chemotherapy should be considered, and surgical treatment is reserved for patients who presented clinical response or who have stable disease after neoadjuvant chemotherapy (interval cytoreduction)^7^ ^-^ ^13^.

Among palliative treatments, there are surgical procedures not intended to increase survival, but rather to increase the quality of life. These are surgeries performed for histopathological confirmation of the disease (laparotomy and/or laparoscopy diagnostic) and cytoreductive surgeries that did not reach the goal of eliminating or reducing the intra-abdominal tumor mass for lesions smaller than 1cm (suboptimal cytoreduction)^7^ ^-^ ^13^.

Cytoreductive surgeries have been indicated for the treatment of patients with ovarian cancer in advanced stages since 1975 when Griffiths et al. evidenced that there was an inverse relationship between residual tumor size and patient survival^14^.

Literature data show that the extent of debulking is correlated with disease-free survival and overall survival^15^ ^,^ ^16^. However, patients with very extensive carcinomatosis and a large volume of disease in the upper abdomen and/or mesenteric involvement tend to obtain lower benefits when undergoing complete and/or optimal debulking procedures^17^ ^,^ ^18^.

## MATERIALS AND METHODS

This is an observational, retrospective study of quantitative analysis data collection through the analysis of medical records. There was no interference in surgical procedure choice, but we were interested in understanding the flow and results of ovarian cancer patients at our UNACON to find faults and propose improvements. Data were collected at a Comprehensive Oncology Unit (UNACON) of the Hospital Foundation of the State of Minas Gerais (FHEMIG), located in Belo Horizonte, accredited by the Brazil Secretary of Health as a reference for oncology care in the state of Minas Gerais.

This is a convenience temporal sample population consisting of all patients diagnosed with malignant ovarian neoplasm with advanced-stage disease, admitted between January 1, 2014, and December 30, 2020, and who underwent cancer treatment in this hospital, to evaluate how surgical treatment impacted the outcomes.

The key objective was to evaluate the quality of surgical treatment mainly concerning its oncological radicality including the types of surgical treatments performed, and their respective percentage were evaluated. The types of surgeries performed were grouped into the following groups: complete cytoreductive surgery (debulking); optimal cytoreductive surgery (debulking); cytoreductive surgery (debulking) suboptimal; and biopsy surgery. The first two were considered adequate and the last two were considered palliative.

Subsequently, patients were stratified into two groups to assess the impact of adequate surgery on these patients’ survival.

- Group 1: Patients who received adequate surgical treatment at some stage of their treatment.
- Group 2: Patients who did not receive adequate surgical treatment at any stage of their treatment.

Data related to the clinical and demographic characteristics of the patients, clinical and/or surgical oncological treatment performed, histopathological results of the surgical specimens, and the follow-up and outcome of the patients after the surgical treatment were collected.

Data were presented in frequency tables with absolute frequencies and their respective percentages to compare the quality of surgical oncological and its oncological radicality when possible, as well as descriptive measures (mean and standard deviation) for quantitative data. To compare categorical variables, the chi-square test and Fisher’s test were used. The overall and disease-free survival curves were done using the Kaplan-Meier method and the comparison of the curves using the Log-Rank test. Variables with at least 80% of the total number of observations were selected for the Cox multivariate regression model. Variables with a p-value <0.20 were selected to compose the initial multivariate logistic model (full). Those variables that did not meet the selection criteria were also evaluated (<0.20) and considered important variables associated with the survival of advanced epithelial ovarian cancer. The Hazard Ratio (HR) was used as a measure of association. The “Backward Method” used was the complete model with successive discarding of the variables that, adjusted concerning the others, did not present a significance level of <0.05. In the evaluated model, no variable was associated, therefore, the initial model was maintained. In all tests, the significance level adopted was 5%, therefore, comparisons whose p-value was less than or equal to 5% were considered significant. The software used for the analysis was SPSS version 23.0. 

## RESULTS

Initially, 92 patients diagnosed with ovarian tumors were identified. One was excluded because of cancer treatment out of research institution, totaling a sample of 91 patients, being 68 patients with advanced ovarian cancer. The following tables show the demographic and clinical characteristics of the patients and the type of primary surgical treatment performed (Tables 1 and 2).

**Table 1:** Clinical characteristics of the study population.

	n	%
Age (Years)*	61.5 ± 12.6 (22 - 88)	
Comorbidities		
Arterial hypertension	49	53.8
Diabetes mellitus	12	13.2
Dyslipidemia	10	11.0
Hypothyroidism	4	4.4
Heart disease	4	4.4
Others	3	3.3
No Comorbidities	31	34.1
History of other neoplasms		
Yes	8	8.8
Not	83	91.2
Performance Status		
PS 0	20	22.0
PS 1	50	54.9
PS 2	4	4.4
PS 3	5	5.5
PS 4	2	2.2
Not reported in the medical record	10	11.0
Surgical Risk		
	n	%
ASA I or RCRI Score 0	7	7.7
ASA II or RCRI Score 1	39	42.9
ASA III or RCRI Score 2	7	7.7
Not reported in the medical record	38	41.8
Staging (FIGO)		
IA	5	5.5
IB	1	1.1
IC	12	13.2
IIA	3	3.3
IIB	3	3.3
IIIA	3	3.3
IIIC	55	60.4
IVB	7	7.7
No report in the medical record	2	2.2
Histopathological Diagnosis		
Ovarian epithelial	78	85.7
Germ Cell Tumors	3	3.3
Stromal Tumors	5	5.5
Carcinosarcoma	3	3.3
Others	2	2.2
Histopathological subtypes of epithelial ovarian carcinoma (n=78)		
Serous	52	66.7
Mucinous	5	6.4
Endometrioid	3	3.8
Clear cells	1	1.3
No report in the medical record	17	21.8
Tumor differentiation degree from epithelial ovarian carcinoma (n=78)		
Well-differentiated	1	1.3
Moderately differentiated	9	11.5
Undifferentiated and/or High Grade	43	55.1
No report in the medical record	25	32.1

ASA: American Society of Anesthesiologists RCRI: Revised Cardiac Risk Index.

**Table 2:** Types of primary surgical treatments performed in patients with advanced ovarian cancer.

	Total	Surgical treatment performed at the institution	Surgical treatment performed at another institution	p-value
				
Oncological surgical treatment considered adequate	5 (7.35%)	4 (7.54%)	1 (6.67%)	0.908^Q^
	Total	Surgical treatment performed at the institution	Surgical treatment performed at another institution	p-value
Oncologic surgical treatment considered palliative	63 (92.65%)	49 (92.45%)	14 (93.33%)	
Total	68 (100%)	53 (100%)	15 (100%)	

^Q^Chi-Square test.

After primary surgical treatment, 63 patients who did not undergo adequate primary surgical treatments were referred for neoadjuvant chemotherapy, to perform interval surgical treatment. At this time of treatment, three patients were lost: two due to death and one due to loss of follow-up.

Of the 60 patients who underwent neoadjuvant chemotherapy, the tumor response to chemotherapy and the type of surgical treatment performed after chemotherapy were evaluated, and the results are described in the tables below (Tables 3 and 4).

**Table 3:** Tumor response after neoadjuvant chemotherapy.

Response to Neoadjuvant Chemotherapy	n	%
Complete response	4	6,7
Partial response	26	43.3
No response	3	5.0
Disease progression	27	45.0
Total	60	100

**Table 4:** Types of surgical treatments performed after chemotherapy.

Types of Surgical Treatments:	n	%
Oncological surgical treatment considered appropriate:		
Complete cytoreductive surgery	8	34.8
Optimal cytoreductive surgery	8	34.8
Oncological surgical treatment considered palliative:		
Suboptimal cytoreductive surgery and/or biopsy	7	30.4
Total	23	100.0

Survival analyzes were performed only for patients with advanced epithelial ovarian cancer, totaling 61 patients, after neoadjuvant chemotherapy who received adequate surgical treatment (68.8%) or palliative surgical treatment (31.2%).

**Figure 1:**
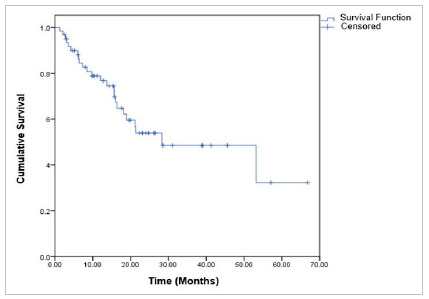
Overall survival for patients with advanced-stage ovarian epithelial tumor.

Median overall survival was 28.3 ± 11.2 months, (95% CI: 6.4 - 50.2). In 12 months, it was 76.7%, in 24 months: 53.9%, in 36 and 48 months: 48.5% and in 60 months 32.3% (Figure 1).

**Figure 2:**
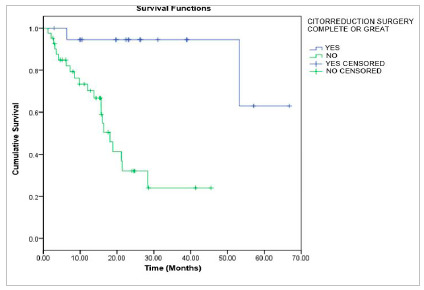
Overall survival for patients with advanced-stage ovarian epithelial tumor, according to the type of surgical treatment performed, p<0.001; log-rank.

There was a statistically significant difference when comparing overall survival according to the type of surgery performed (p<0.001). Patients who underwent adequate surgery had better overall survival than those who did not undergo this type of surgery. The overall survival at 12, 24, 36, and 48 months was 94.4% and at 60 months 63%; against one in 12 months of 70.2%, 24 months of 32.2%, and in 36 months of 24.1%, not reaching follow-up at 48 and 60 months; respectively (Figure 2).

There was no significant difference in overall survival stratified by performance status (PS), surgical risk, degree of differentiation, and surgeon specialty (Figures 4-6).

**Figure 3:**
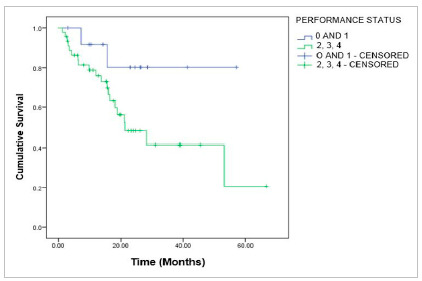
Overall survival for patients with advanced-stage ovarian epithelial tumor, according to Performance Status (PS), p=0.062; log-rank.

**Figure 4:**
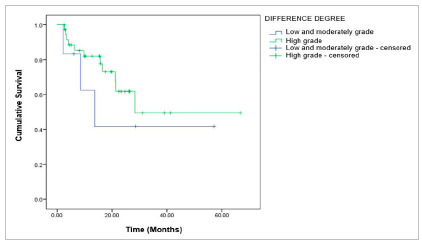
Overall survival for patients with advanced-stage ovarian epithelial tumor, according to the Degree of Differentiation, p=0.392; log-rank.

**Figure 5 f5:**
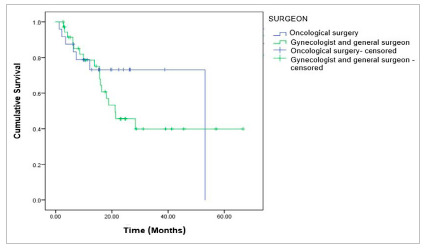
Overall survival for patients with advanced-stage ovarian epithelial tumor, according to the surgeon’s specialty, p=0.437; log-rank.

**Figure 6:**
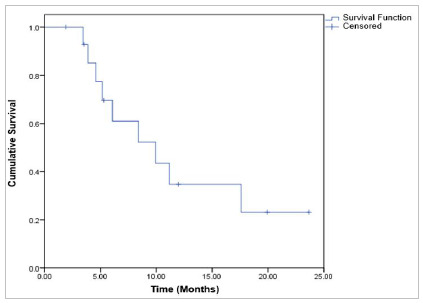
Disease-free survival for patients with advanced-stage ovarian epithelial tumor.

No combined factors were found to be associated with overall survival in the Cox multivariate model (Table 6).

The median disease-free survival of patients with advanced-stage ovarian epithelial tumor (FIGO stage IIB-IV) was 9.9 ± 3.2 months, (95% CI: 3.7 - 16.1). The 12-month disease-free survival was 34.8% and the 24-month survival was 23.2% (Figure 6).

## DISCUSSION

When evaluating the type of primary surgical treatment performed in patients with advanced-stage ovarian cancer, we observed as shown in Table 3 that only five out of a total of 68 patients (7.35%) underwent an oncological surgical treatment considered adequate up-front, showing a large discrepancy about literature data that consider acceptable rates of around 50%. In reference centers for the treatment of ovarian cancer, these rates can reach levels of 70 to 80%^7^ ^-^ ^11^.

One hypothesis that could be raised to justify these data would be the fact that our patients are being referred to neoadjuvant treatment because they have very extensive disease, during primary surgery, which would make it impossible to perform a complete or optimal cytoreductive surgery. This hypothesis can be supported as from 68 patients, 55 were stage IIIC and seven were stage IVB (91.2%), which means a very extensive disease that could have led the surgical team to decide on neoadjuvant chemotherapy and further interval surgical treatment^7^ ^-^ ^13^. Those differences were not related to surgical specialty or the competence of the surgical team but mainly to tumor patients staging presenting for surgery with advanced disease when is not feasible to perform standard patterns of oncological procedures. It was not possible to consider if this advanced stage disease at diagnosis was related to bad tumor biology or a long time to have confirmed ovarian cancer diagnosis.

At this point, there is a new question: What are the reasons that led to only 38.3% of patients referred to neoadjuvant chemotherapy having undergone interval surgical treatment, as the literature shows rates of approximately 90%? One hypothesis that can be raised to explain these data is the disease progression rates (45%) observed during neoadjuvant chemotherapy much higher than those presented in multicenter studies (10%). This factor may have prevented interval surgical treatment in a large percentage of patients^7^ ^-^ ^13^, but with the data evaluated in this study, we cannot define the real reason that led to these high rates of disease progression during neoadjuvant therapy.

Despite the low rates of patients undergoing interval surgical treatment, this group of patients presented the best rates (69.6%) of surgeries considered adequate corroborating the rates from reference centers in the treatment of ovarian cancer. A plausible explanation for these findings is that these patients, after chemotherapy treatment, may have been followed up and surgically treated by experienced professionals with more specific training in high-complexity surgeries, such as multiorgan resections^7^ ^-^ ^13^.

However, when analyzing the institution in totality, the total number of patients diagnosed with advanced-stage ovarian cancer who underwent surgical oncological treatment considered adequate, whether performed primarily or after chemotherapy, was only 30.9%, which is, much lower than the rates reported in the literature for reference centers for the treatment of ovarian cancer^7^ ^-^ ^11^.

To analyze the impact of surgical treatment on the prognosis of the studied patients and facilitate comparison with the literature, we selected only patients with advanced-stage ovarian cancer, whose histopathological classification was of the epithelial type.

In this group of patients, we found a median overall survival of 28.3 ± 11.2 months, (95% CI: 6.4 - 50.2). The 5-year overall survival was 32.3%, slightly below those found in the literature for patients in FIGO stages IIIC, which represented 82% of our sample^8^. A statistically significant difference was found when comparing overall survival according to the type of surgery performed (p<0.001). Patients who underwent adequate surgery achieved 94% of overall survival (12 to 48 months) and 63% (60 months) compared to patients who did not undergo adequate surgery: 70.2% (12 months), 32.2% (24 months), 24,1% (36 months), and without reaching a 48 and 60-months follow-up. Cancer treatment carried out in reference hospitals for the treatment of ovarian cancer and by a well-trained multi-disciplinary team, especially by an experienced surgical team capable of performing highly complex procedures such as surgeries that require multiorgan resections, positively influences surgical treatment rates adequate.

## CONCLUSION

Overall survival of advanced-stage epithelial ovarian cancer patients is directly influenced by appropriate surgical treatment. However, in the present study, the percentage of patients receiving the adequate surgical treatment was much lower than the rates reported in the literature. We believe that these surgically undertreated patients are not related to the specialty of the surgical team nor its surgical competence, but mainly to the patient’s late diagnosis before reaching the oncological reference center. A basic solution to this problem would be qualified surgeons in oncological gynecology following ovarian cancer patients from diagnosis until after receiving neoadjuvant chemotherapy, to avoid missing the right window to perform adequate surgery. The definitive solution would be a clinical team working closer to surgical teams, and keeping in mind that ovarian cancer patients could have their overall survival strongly improved if they receive optimal surgical oncological treatment once on their oncological journey.
